# Translation, cross-cultural adaptation, and measurement properties of the Arabic version of the pain sensitivity questionnaire

**DOI:** 10.3389/fpain.2024.1339449

**Published:** 2024-02-06

**Authors:** Abdullah Alqarni, Fayaz Khan, Umar Alabasi, Ruth Ruscheweyh

**Affiliations:** ^1^ Department of Physical Therapy, Faculty of Medical Rehabilitation Sciences, King Abdulaziz University, Jeddah, Saudi Arabia; ^2^ Department of Neurology, University of Munich, Munich, Germany

**Keywords:** central sensitization, pain sensitivity questionnaire, Arabic translation, cultural adaptation, psychometric properties

## Abstract

**Background:**

The Pain Sensitivity Questionnaire (PSQ) is a reliable and valid self-reported tool for the assessment of pain sensitivity in clinical practice. The PSQ has been translated, validated, and cross-culturally adapted into multiple languages. However, a validated Arabic version of the PSQ is not available. Thus, this study aims to translate, validate, and cross-culturally adapt the English version of the PSQ into the Arabic language.

**Methods and materials:**

The English version of the PSQ was translated and culturally adapted into Arabic following international guidelines. The psychometric properties of the final version of the PSQ-Arabic (PSQ-A) were tested among 119 patients with different persistent musculoskeletal (MSK) pain.

**Findings:**

The Cronbach’s α for the PSQ-A-total, PSQ-A-moderate, and PSQ-C-minor were 0.81, 0.79, and 0.76, respectively. The means for the PSQ-A-total, PSQ-A-moderate, and PSQ-C-minor scores were 5.07 (±1.28), 5.64 (±2.07), and 4.50 (±0.50). The test-retest reliability measured with the interclass correlation coefficient for 68 subjects was 0.80 for the PSQ-A-total, 0.74 for the PSQ-A-moderate, and 0.77 for the PSQ-A-minor. The PSQ-A-total and the PSQ-A-minor showed positive significant correlations with pain catastrophizing scale (PCS) (*r* = 0.15, 0.17); *P* ≤ 0.05), respectively. The PSQ-A-total, PSQ-A-moderate, and PSQ-A-minor showed positive significant correlations with the Brief Pain Inventory (BPI)-pain scores (*r* = 0.47, 0.43, 0.45; *P* ≤ 0.01), respectively and with the BPI-pain interference scores (*r* = 0.37, 0.33, 0.34; *P* ≤ 0.01), respectively.

**Conclusions:**

This study shows that the PSQ-A is a reliable and valid tool to assess individuals with pain sensitivity in Arabic populations. Further studies are recommended to examine the concurrent validity of the PSQ-A against experimental pain sensitivity measures.

## 1 Introduction

Persistent pain is a global burden, affecting up to a quarter of the global population, and has a massive effect on economic and healthcare systems (1, 2). In particular, persistent musculoskeletal (MSK) pain is considered one of the most common causes of years lived with disability (3–5). Persistent MSK pain refers to “pain in muscles, tendons, joints, and ligaments for more than three months (6, 7). Persistent MSK pain is largely affected by the central nervous system including peripheral and central sensitization, reduced anti-nociception, increased pro-nociception, and alteration of cortical pain processing (8–10). The International Association for the Study of Pain defines central sensitization as “increased responsiveness of nociceptive neurons in the central nervous system to either normal or subthreshold afferent input” (11). Several persistent MSK pain conditions showed evidence of central sensitization/elevated pain sensitivity, such as low back pain (12), neck pain (13), knee osteoarthritis (14), shoulder pain (15, 16). In an attempt to assess altered pain sensitivity, multiple test procedures have been proposed in the literature, such as quantitative sensory testing (QST) procedures.

The QST test procedures are psychophysical experimental tests designed to measure the pain threshold to controlled sensory stimuli (17). However, the QST test procedures are time-consuming, require a battery of specialized expensive tools, and standardized protocols (18). Therefore, there is a need for an alternative simple, easy to administer, and less time-consuming testing measure for pain sensitivity. The Pain Sensitivity Questionnaire (PSQ) has a potential advantage in clinical settings for assessing pain sensitivity (19). The PSQ is a reliable and valid self-reported questionnaire, which was developed to assess a patient's perception various imagined physical stimuli occurring in daily life (20).

The English version of the PSQ has shown associations with a variety of QST, including pain threshold and suprathreshold responses, in healthy individuals and patients with chronic pain conditions (20–23). The PSQ has been translated, validated, and cross-culturally adapted into multiple languages, such as English (22), Norwegian (23), Polish (24), French (25), Dutch (26), Mandarin Chinese (27), Iranian (28), and Turkish (29). The previous studies indicated that the PSQ could be utilized in research and clinical sitting.

To date, the PSQ has not been translated into the Arabic language. Hence, translation and adaptation of the PSQ into Arabic will assist in assessing many patients with chronic pain in Saudi Arabia and other countries using Arabic as a spoken language for providing better assessment and management strategies. Therefore, this study aims to translate, validate, and cross-culturally adapt the English version of the PSQ into the Arabic language.

## 2 Materials and methods

### 2.1 Translation and cross-cultural adaptation

The English version of the PSQ was translated into Arabic according to Wild et al. (30) and Beaton (31) recommendations as follows; permission was sought from the original author of the PSQ (20) to translate the English version of the PSQ into Arabic. This was followed by a forward translation of the English version of the PSQ into Arabic by two native Arabic (a medical practitioner, and a non-medical practitioner) who are ﬂuent in both English and Arabic. Both translators independently translated the English version of the PSQ into Arabic. One of the translators was aware of the purpose of the PSQ translation, while the other was not. Then, the two translators and the research team combined the two Arabic versions into a single Arabic version. Backward translation was conducted by two professional translators (One with a medical background and one who has no experience in using medical terminologies) who were fluent in both English and Arabic languages. Both translators were not aware of the purpose of translation and were not aware of the English version of the scale. An expert committee [previous four translators involved in the process, the developer (Ruscheweyh) of the English version of the PSQ, and an Arabic translation expert] discussed the two back-translated versions of the PSQ and the English version. Then, the committee evaluated the semantic, idiomatic, experiential, and conceptual equivalence of all items and answered until a consensus was achieved on the pre-ﬁnal version of PSQ. The pre-ﬁnal version was piloted among 30 participants for cognitive debriefing/face validity. Participants were asked for opinions about the understanding of the wording and clarity of the pre-final version. The committee approved the pre-final version without amendments.

### 2.2 Validation of the PSQ-Arabic

#### 2.2.1 Study design

Cross-sectional observational study. The study was approved by the Centre of Excellence in Genomic Medical Research, King Abdul-Aziz University, Jeddah, (Reference: 10-CEGMR-Bioeth-2020).

#### 2.2.2 Participants and setting

This study was conducted at the outpatient clinic of the department of physical therapy at the faculty of applied medical sciences, King Abdulaziz University. The inclusion criteria for participants were a native Arabic who speaks and reads the Arabic language, an adult (aged ≥18 years) with persistent MSK pain (>3months), and has no cognitive impairments. Participants were excluded if they have a fever or infectious disease (e.g., Covid-19) at the time of participation in the study, psychiatric disorders or neurological diseases (e.g., stroke, hemiparesis, or epilepsy), or used any painkillers within the past 24 h. Informed consent was obtained from the subjects at recruitment. Participants completed the Brief Pain Inventory-Arabic (BPI-Arabic) (32), Hospital Anxiety and Depression Scale-Arabic (HADS-Arabic) (33), Pain Catastrophizing Scale-Arabic (PCS-Arabic) (33), and the PSQ-Arabic.

#### 2.2.3 Pain sensitivity questionnaire (PSQ)

The PSQ is based on an individual’s rating of pain intensity in response to 17 imagined daily life painful situations (20). Respondents score their pain intensity on a numerical rating scale (NRS) of 0 to 10, with (0) indicating no pain at all and (10) indicating the worst pain imaginable. The PSQ-total has two subscales (PSQ-moderate and PSQ-minor) of seven items. The PSQ-total is the average rating of items (1–4, 6–8, 10–12, 14–17). The PSQ-moderate subscale score is the average rating of items (1–3, 8, 15–17) indicating moderate pain, while PSQ-minor is the average rating of items (3, 6, 7, 10–12, and 14) indicating minor pain. Three items (5, 9, and 13) are not included in the scores as these items are directed to normally non-painful situations.

#### 2.2.4 Brief pain inventory-Arabic (BPI-Arabic)

The Brief Pain Inventory (BPI) is designed to assess patients’ pain intensity and pain interference (34). Pain severity is measured with four items: worst pain in the last 24 h, least pain in the last 24 h, pain on average, and pain right now. The intensity of pain is rated using a 0–10 rating scale anchored at zero (no pain) to 10 (pain as bad as you can imagine). Pain interference is measured with seven domains of functioning including general activities, mood, walking ability, normal work, relations with others, sleep, and enjoyment of life. Patients rated from 0 (does not interfere) to 10 (completely interferes). This study adopted the Arabic version of the BPI. An Arabic version of the BPI was available and Cronbach's alpha coefficients were reported as 0.82 and 0.92 for the severity and interference items, respectively. Factor analysis yielded two factors and the correlations between the severity and interference items ranged between 0.25 and 0.57 (*P* < 0.05) (32).

#### 2.2.5 Hospital anxiety and depression scale—Arabic

Zigmond and Snaith (35) identified the original HADS for measuring anxiety and depression disorders among patients in general clinics. The HADS consists of 14 items: anxiety (7-item) and depression (7-item). These items are rated on a 4-point scale (0 = absence of symptoms, 3 = maximum symptoms). The scores for each subscale range from 0 to 21, with a score of 0–7 is considered normal, 8–10 (mild), 11–14 (moderate), and 15–21 (severe). The entire scale ranges from 0 to 42, with higher scores indicating a higher level of emotional distress. This study adopted the Arabic version of HADS. The Cronbach’s *α*s for the HADS anxiety subscale were 0.83 (95%) confidence interval (0.79–0.88), and for the HADS depression subscale were 0.77 (0.7–0.83). HADS anxiety score was strongly correlated (*r* = 0.67) with generalized anxiety disorder 7-item scale, and the HADS depression score was strongly associated (*r* = 0.66) with the major depression inventory (36).

#### 2.2.5 Catastrophizing scale—Arabic

Sullivan et al. (37) developed the PCS, which contains 13 items assessing the thoughts and feelings associated with pain. The PCS includes three subscales: Rumination, magnification, and helplessness. The PCS items are rated on 5-points scale (0 = not at all, 1 = to a slight degree, 2 = to a moderate degree, 3 = to a great degree, 4 = all the time). The higher PCS score indicates a higher tendency to pain catastrophizing. Arabic version of the PCS was available and a Cronbach's alpha of 0.94 was reported, test-retest reliability (*r* = 0.84). This study adopted the Arabic version of PCS (36).

### 2.3 Statistical analysis

#### 2.3.1 Sample size

A power analysis was conducted using G Power software (version 3.1.2; Kiel, Germany) to determine the number of participants included in the study. Assuming correlations for PSQ and Pain Catastrophizing scale to be 0.3 (moderate reference), power of 95 and ∝ error as 0.05, resulted in a minimum sample size of 111.

### 2.4 Descriptive analysis

All statistical analyses were performed using SPSS 20.0 statistics package (SPSS, Inc., Chicago, IL, U.S.A.). Demographic and clinical characteristics of the sample were analyzed using frequencies, means, and standard deviations (SDs). Questionnaires with missing items in any scales were excluded from the analysis.

### 2.5 Inferential analysis

#### 2.5.1 Reliability

##### 2.5.1.1 Internal consistency

The Cronbach’s alpha was used with a value of 0.60 indicating acceptable internal consistency and more than 0.70 indicating good internal consistency.

##### 2.5.1.2 Test-retest reliability

The ﬁnal PSQ—Arabic version was assessed on two occasions, which were separated by two weeks. Reliability was analyzed using the intraclass correlation coefficient (ICC). The values of ICC were indicated as excellent at 0.8, moderate from 0.6 to 0.79, and poor at 0.61.

#### 2.5.2 Validity

##### 2.5.2.1 Floor and ceiling effects

This was measured as the per cent of patients who reported a minimum or maximum score of PSQ-minor, PSQ-moderate, and PSQ-total. The desired value for the floor/ceiling effect is less than 15% to 20%.

##### 2.5.2.2 Convergent validity

Pearson's correlation was utilized according to the normality of the data for comparing the results of the PSQ-A with the results of the validated Arabic version of the PCS-A, the Brief Pain Inventory-A, and the Hospital Anxiety and Depression Scale-(HADS)-Arabic. The correlation coefficient was considered strong if it was greater than 0.50, and moderate between 0.30 and 0.50.

##### 2.5.2.3 Construct validity

A confirmatory factor analysis (CFA) was conducted using AMOS software. This analysis showed the correlation between PSQ-A items and the PSQ-A subscales (minor and moderate). The model-fit indices included chi-square (*χ*^2^), comparative fit index (CFI), and root mean square error of approximation (RMSEA). For RMSEA, values of 0.08 or below indicate a close fit, and values in the range from 0.08 to 0.10 indicate an acceptable fit.

## 3 Results

### 3.1 Participants

A total of 119 patients with different persistent MSK pain (neck (*n* = 22), back (*n* = 60), shoulder (*n* = 26), knee (*n* = 8), and ankle (*n* = 3)) participated in this study. Of this sample, 67% (*n* = 80) were female, and the mean age of participants was 39.5 years (Table 1). Among the participants, 58% were not working. The mean for the PSQ-A-total score was 5.07 (±1.28), 5.64 (±2.07) for the PSQ-A-moderate, and 4.50 (±0.50) for PSQ-A-minor.

**Table 1:** Demographic data of subjects.

Characteristics		Percentage (%)/count (*n*)
Age (Years, mean ± SD)		39.5 (±2.5)
Gender	Male	33% (39)
	Female	67% (80)
Work status	Working	42% (50)
	Not working	58% (69)
Area of pain	Neck	18.48% (22)
	Back	50.42% (60)
	Shoulder	21.84% (26)
	Knee	6.72% (8)
	Ankle	2.52% (3)
PSQ-A-total (mean ± SD)		5.07 (±1.28)
PSQ-A-moderate (mean ± SD)		5.64 (±2.07)
PSQ-A-minor (mean ± SD)		4.50 (±0.50)

### 3.2 Floor and ceiling effects

For the PSQ-A-total and the minor scores, the percentage of participants who scored the minimum and the maximum was 0.84%. For the PSQ-A-moderate score, the percentage of participants who scored the minimum was 0.84%, and the maximum was 3.36%.

### 3.3 Internal consistency

The Cronbach's α for the PSQ-A-total was 0.81, 0.76 for PSQ-A-minor, and 0.79 for PSQ-A-moderate.

### 3.4 Test-retest reliability

The first 68 subjects participated in a retest assessment after two weeks. The intraclass correlation coefficient was 0.80 for the PSQ-A-total, 0.74 for the PSQ-A-moderate, and 0.77 for the PSQ-A-minor.

### 3.5 Convergent validity

The PSQ-A-total and the PSQ-A-minor showed positive significant correlations with the pain-specific measure PCS at (*P* ≤ 0.05). In addition, the PSQ-A-total and the two PSQ-A subscales showed positive significant correlations with the BPI-pain score and BPI-pain interference score at (*P* ≤ 0.01). However, there was no significant correlation with the HADS-D or HADS-A (Table 2).

**Table 2:** Correlations between PSQ-A scores, psychological measures, and pain characteristics.

	PSQ-A-total	PSQ-A-moderate	PSQ-A-minor
PCS	0.154*	0.110	0.169*
HADS-D	−0.110	−0.159	−0.054
HADS-A	−0.019	−0.055	0.011
BPI pain score	0.473**	0.428**	0.443**
BPI interference score	0.365**	0.331**	0.342**

* Pearson's correlation coefficient is significant at (*P* ≤ 0.05).

** Pearson's correlation coefficient is significant at (*P* ≤ 0.01).

### 3.6 Construct validity

**Figure 1:**
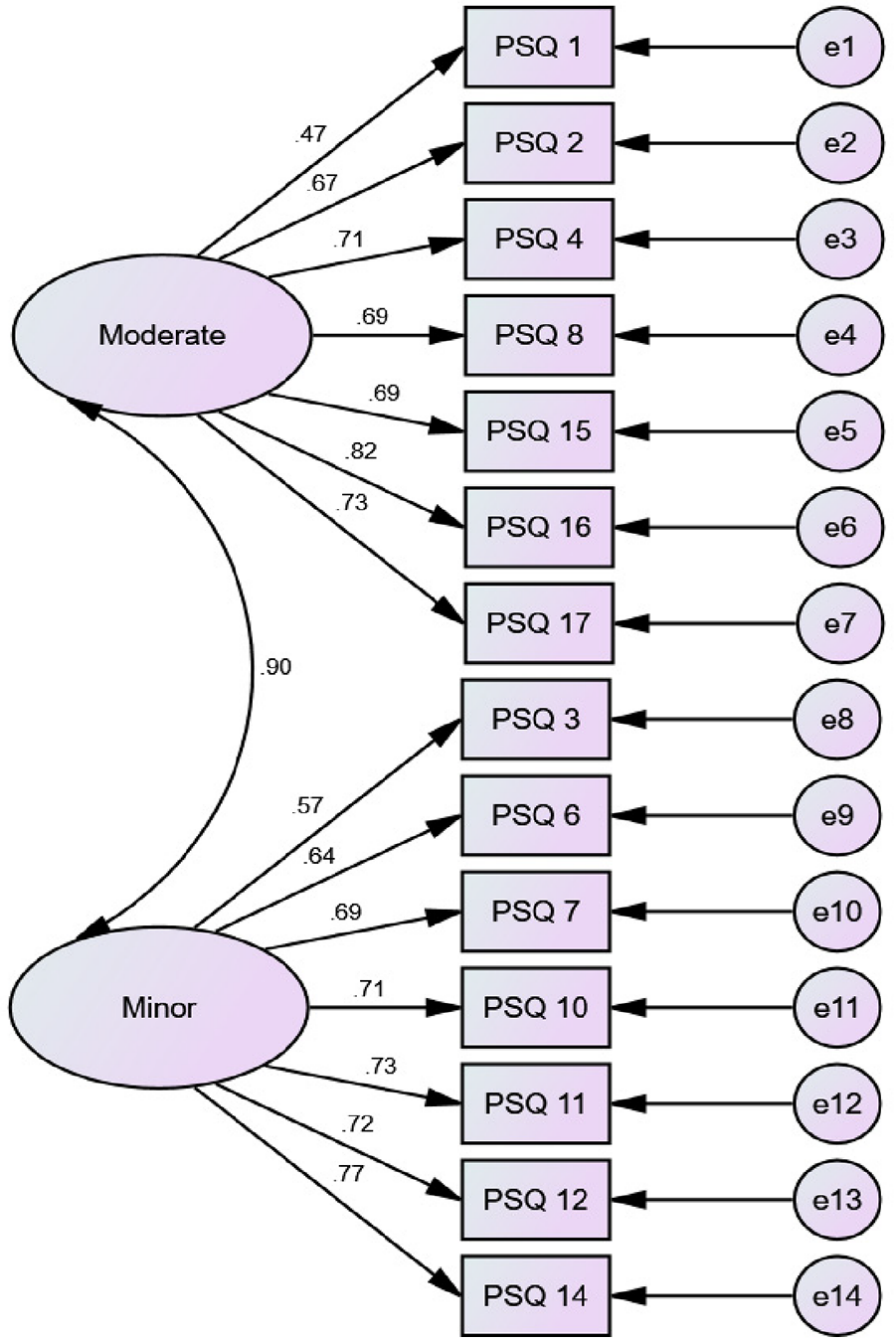
Factor structure of PSQ-A.

According to Ruscheweyh et al. (20), a 2-factor model for the PSQ-A was built: the PSQ-A-moderate (7-item factor) and the PSQ-A-minor (7-item factor). An acceptable model fit was achieved: Chi-Square/Degree of Freedom (CMIN/DF) = 3.33, Goodness of fit Root (GFI) = 0.85, Comparative Fit Index (CFI) = 0.87, Root Mean Square Error of Approximation (RMSEA) = 0.11. Correlations between items within the same factor were shown in Figure 1.

## 4 Discussion

This study aimed at cross-cultural adaptation, reliability, and validity of the PSQ-A. The finding from this study indicated that the PSQ-A is an easy to understand, administered, reliable, and valid measure of pain sensitivity in individuals with persistent MSK pain. The internal consistency of the PSQ-A-total, PSQ-A-moderate, and PSQ-A-minor was good; the Cronbach's α were 0.81, 0.76, and 0.79, respectively. These were similar to Cronbach's alphas of the Chinese version (0.90, 0.86, and 0.81) (27), the German PSQ version (0.92, 0.91, and 0.81) (20), the Korean version (0.93, 0.88, and 0.87) (38), the Iranian version (0.81, 0.82, and 0.82) (28), the Dutch version (0.90, 0.86, and 0.82) (26), the Norwegian version (0.92, 0.90, and 0.85) (23), and the Spanish version (0.95, 0.91, and 0.92) (39). Confirmatory factor analysis confirmed the two-factor structure of the PSQ-A with the two subscores PSQ-A minor and PSQ-A moderate to be consistent with the original description (20).

The test-retest reliability measured with the interclass correlation coefficient (ICC) was 0.80 for the PSQ-A-total, 0.74 for the PSQ-A-moderate, and 0.77 for the PSQ-A-minor. These values were similar to the values for the Korean version, which were 0.78, 0.79, and 0.75 (38), the Chinese (0.73, 0.74, and 0.68) (27), but slightly lower than those for the German PSQ version (0.83, 0.79, and 0.86) (20), the Polish version [(0.93, 0.87, 0.91) (24)], the Iranian version (0.84, 0.84, and 0.85) (28), and the Spanish (0.84, 0.82, and 0.84) (39).

The PSQ-A-total and the PSQ-A-minor showed weak positive significant correlations with the pain-specific measure PCS (*r* = 0.15, 0.17), respectively. This may due to the large proportion of female participants (67%, *n* = 80) in this study who reported higher level of catastrophizing related pain than male. This is not surprising, as it has been reported in the literature that female would show higher levels of catastrophizing than male (37). In addition, altered pain sensitivity linked with increased catastrophizing. Meints et al. (40), reported that there is association between catastrophizing and sensitization which resulted in an increase of clinical pain among individuals with chronic LBP*.* Our results were similar in the magnitude of correlation for the previous translated versions including the Chinese (*r* = 0.27, 0.27) (27), German (*r* = 0.45, 0.38) (20), English (*r* = 0.32, 0.33) (22), and Korean (*r* = 0.38, 0.37) (38). The Spanish version of PSQ-total and PSQ-minor showed a stronger positive correlation with the PCS at (0.58, 0.60) (39) and Iranian version for the PSQ-total score (*r* = 0.81) (28). On the contrary, the Turkish version of the PSQ-total and subscales did not correlate with the PCS (29). The PSQ-A-moderate did not correlate with the PCS; the other translated versions were positively correlated. Moreover, the PSQ-A-total and both subscales did not significantly correlate with the HADS-D or the HADS-A. These findings were similar to the English and Turkish versions of the PSQ (22, 29). This may due to the fact that the PSQ is based on an individual's rating of pain intensity in response to imagining situations, which more directly measure the sensory facilitation involved in CS (20), however the degree to which PSQ reflects the top–down pain mechanisms related to psychological factors remain open (41). Recent systematic review and meta-analysis indicated that the psychological measures of depression and anxiety including the HADS-D and the HADS-A showed a weak correlation with the PSQ (*r* = 0.11, 0.16, respectively), while pain catastrophizing showed a moderate correlation with the PSQ (*r* = 0.32) (41). Accordingly, correlations between PSQ-A and the pain-specific measure PCS were higher than correlations between PSQ-A and HADS in the present study.

The PSQ-A-total, PSQ-A-moderate, and PSQ-A-minor showed positive significant correlations with the BPI-pain scores (*r* = 0.47, 0.43, 0.45, respectively) and with the BPI-pain interference scores (*r* = 0.37, 0.33, 0.34, respectively). The previous findings were similar to the Turkish version (29), which showed a similar magnitude of correlations for the BPI-pain scores (*r* = 0.28, 0.31, 0.24, respectively) and with the BPI-pain interference scores of the total and minor subscale (*r* = 0.31, 0.34, respectively). On the other hand, the PSQ-total score of the English version was the only score significantly correlated with the BPI-pain score (*r* = 0.25) (22), while the correlations with the BPI interference score did not reach significance for the PSQ-E-total and both subscales.

The PSQ-A scores were similar to those found in previous studies. The mean of PSQ-A-total scores was 5.07, while for the Korean version was (5.93) (38), Chinese (4.7) (27), and Norwegian (4.5) (23). However, the PSQ-A-total was higher than those reported in the original study (4.0) (20), the Dutch version (4.1) (26), and the English version (3.6) (22). The mean of the PSQ-A-moderate score was 5.64, which was similar to other versions, which range from 4.7 to 6.5 (20, 22, 23, 26, 27, 38). The mean of the PSQ-A-minor score was 4.4, which is higher than the English version (2.5) (22) and other European versions, such as the German version (2.5) (20), Dutch (2.8) (26), and the Norwegian version (3.1) (23). In the contrary, the PSQ-A-minor score was similar to the Asian versions, such as the Chinese version (3.9) (27) and the Korean version (5.4) (38). The discrepancies in scoring the PSQ-minor may be related to the ethnicity or cultural influence of reporting pain sensitivity (42). In an experimental pain sensitivity, Asians demonstrated significantly lower pain threshold and tolerance levels than Whites (43). In addition, Asians report a higher widespread musculoskeletal pain than Whites (44, 45).

## 5 Limitations

This study had some limitations as it included a higher number of female participants, which might inflate the PSQ-A scores. Evidence from the literature revealed that females report higher pain sensitivity than males (46). Another possible limitation of the PSQ-A is that one question asks participants about snow (item 12), which makes it less applicable to countries with a warmer climate. Furthermore, the Arabic version of the PSQ has translated the English version of the same construct rather than the original German version.

## 6 Conclusion

This study demonstrated that the Arabic version of the PSQ is a reliable and valid tool to assess pain sensitivity in individuals with persistent MSK pain. Therefore, this tool can be used to assess pain sensitivity in clinical practice. Further studies are needed to examine the concurrent validity of the Arabic version of the PSQ against experimental pain sensitivity measures, such as QST procedures.

## Data Availability

The raw data supporting the conclusions of this article will be made available by the authors, without undue reservation.
